# Genomic portrayal of emerging carbapenem-resistant El Tor variant *Vibrio cholerae* O1

**DOI:** 10.1128/aac.00740-25

**Published:** 2025-10-17

**Authors:** Sreeja Shaw, Agila Kumari Pragasam, Goutam Chowdhury, Prosenjit Samanta, Deboleena Roy, Debjani Ghosh, Thandavarayan Ramamurthy, Jigna Karia, Govind Ninama, Shin-ichi Miyoshi, Yukihiro Akeda, Hemanta Koley, Asish Kumar Mukhopadhyay

**Affiliations:** 1 ICMR-National Institute for Research in Bacterial Infections 30170 , Kolkata, West Bengal, India; 2 V. Ramalingaswami Bhawan, Indian Council of Medical Research 28604, New Delhi, India; 3 Medical College Baroda 29010, Vadodara, Gujarat, India; 4 SSG Hospital 81276 , Vadodara, Gujarat, India; 5 Okayama University 12997, Okayama, Okayama, Japan; 6 National Institute of Infectious Diseases 13511, Tokyo, Japan; University of Fribourg, Fribourg, Switzerland

**Keywords:** antimicrobial resistance, *Vibrio cholerae*, bla_NDM-1_, carbapenem resistance, horizontal gene transfer, IncA/C plasmid

## Abstract

The escalating prevalence of carbapenem-resistant (CR) enteric pathogens elicits significant challenges to public health management and effective antimicrobial therapy. While carbapenem resistance is rare in *Vibrio cholerae* O1 (VC), the recent emergence of CR strains reveals a concerning shift in their antimicrobial resistance (AMR) landscape. This study aims to characterize the resistance mechanisms in newly identified El Tor CRVC isolated from cholera patients in Gujarat, India during 2019. Fifty VC isolates were screened for major virulence-associated genes along with the determination of their antibiotic resistance profiles using Kirby-Bauer disk diffusion and MIC assays. Whole-genome sequencing (WGS) was employed to investigate the underlying mechanisms of CR. All the isolates exhibited hypervirulent Haitian alleles of major virulence genes and AMR profiles of typical multidrug resistance (MDR). Strikingly, 12% (6/50) of them were resistant to carbapenems and other antibiotics. Molecular analysis revealed that these CR isolates were clonally related and harbored a 142 kbp IncA/C type conjugative mega-plasmid with several AMR encoding genes, including *bla*_NDM-1_, that can be easily transferred to other bacterial species and confer donor AMR patterns. The plasmid’s competence for horizontal gene transfer presents a significant risk of dissemination to other enteric pathogens and thereby may complicate the treatment. This finding emphasizes the urgent need for enhanced genomic surveillance and robust antimicrobial stewardship programs aimed at curbing the spread of CRVC strains and mitigating their impact on cholera treatment and containment strategies.

## INTRODUCTION

Cholera remains a severe global health threat, particularly in regions with inadequate sanitation, overcrowding, flooding, and limited access to clean drinking water. The disease is primarily caused by the ingestion of food and water contaminated with the toxigenic strains of the Gram-negative bacterium *Vibrio cholerae*, specifically the O1 and O139 serogroups (1). Historically, the classical biotype of *V. cholerae* O1 was responsible for the first six cholera pandemics. However, the ongoing seventh pandemic (7P) is driven by the El Tor biotype, which emerged in 1961 and is associated with the longest cholera pandemic. This biotype has undergone various phenotypic and genetic modifications, contributing to its virulence, antimicrobial resistance (AMR), transmission, and global dissemination, leading to 535,321 reported cases of cholera and more than 4,000 deaths (2).

Recently, *V. cholerae* O1 has developed resistance to broad-spectrum antimicrobials, including ampicillin, ciprofloxacin, tetracycline, macrolides, quinolones, trimethoprim/sulfamethoxazole, and even carbapenems, which are considered the last line of defense against multidrug-resistant (MDR) Gram-negative pathogens (3–8). Several findings have highlighted the emergence of MDR *V. cholerae* O1 El Tor driven by mechanisms, such as genetic mutations, the acquisition of extrachromosomal mobile genetic elements (MGEs), and efflux pump-mediated drug expulsion (9–12). The genomic analysis of *V. cholerae* isolates from Yemen in 2019 revealed resistance to macrolides and third-generation cephalosporins, which were previously effective during earlier outbreaks in the same region (7). This resistance was linked to a 139 kbp IncC plasmid containing a 20 kb pseudo-compound transposon, suggesting horizontal gene transfer (HGT) between the isolates (7).

In recent years, carbapenem resistance (CR) in enteric pathogens has become a growing global concern primarily due to the production of carbapenemases. Although carbapenems are not typically the first choice for treating cholera, the increasing prevalence of carbapenem resistance in *Vibrio* spp. over the past decade highlights the role of environmental microbial populations as reservoirs for several other antimicrobial resistance encoding genes (ARGs). The presence of CR-encoded *bla*_NDM-1_ is generally associated with MDR (13). Notably, *V. cholerae*, *V. vulnificus*, and *V. parahaemolyticus* are of significant clinical relevance and have exhibited MDR. Reports of CR are not confined to the Indian subcontinent; such resistance has also been documented in other continents, including the US and Europe. Of the 142 species of the genus *Vibrio*, many have been shown to resist carbapenem. The genetic adaptability of *Vibrio*, coupled with its ability to acquire environmental DNA (eDNA) through HGT, contributes to the dynamics of AMR, and these bacteria are the potential reservoirs of ARGs. Phenotypic susceptibility to imipenem has been reported despite the presence of *bla*_NDM-1_ in *V. parahaemolyticus* (14). Similarly, *V. alginolyticus* harboring metallo-β-lactamase (VAM-1) gene has also been shown to be susceptible to imipenem. These findings defy the results obtained using routine susceptibility assays. Additionally, carbapenemase-producing bacteria may also exhibit resistance to several β-lactams as well as other classes of antimicrobials. Nevertheless, the potential for dissemination of ARGs to other species through HGT remains a significant risk. Given the emergence of carbapenem-resistant *V. cholerae* O1 (CRVC) as an imminent threat, this study aims to conduct a detailed whole genome sequencing (WGS)-based characterization of *V. cholerae* O1 El Tor isolated from Gujarat, India.

## RESULTS

### Phenotypic and molecular characterization of *V. cholerae*

All the tested *V. cholerae* isolates belonged to the serogroup O1, specifically the Ogawa serotype. The production of acetoin in the VP test and visible hemolysis on sheep blood agar plates were observed for all the isolates similar to the El Tor reference strain N16961. However, all the isolates showed sensitivity toward PB, similar to the classical biotype strain O395. Virulence gene profiling revealed that all belong to the hypervirulent Haitian strain alleles of the *ctxB*, *tcpA*, and *rtxA*. The susceptibility patterns of these isolates were assessed using 16 antimicrobials. Most of the isolates exhibited MDR, being resistant to at least three different drugs. All the isolates were resistant to streptomycin and trimethoprim/sulfamethoxazole but susceptible to tetracycline, norfloxacin, ofloxacin, azithromycin, and doxycycline. About 96% of the isolates were resistant to nalidixic acid, and reduced susceptibility was observed for chloramphenicol (76%) and ciprofloxacin (12%) (Fig. S1). A unique AMR profile was identified in 12% (6/50) of the isolates, which showed resistance to ampicillin, ceftriaxone, cefotaxime, ceftazidime, gentamicin, and meropenem (Fig. S1). These isolates exhibited greater minimum inhibitory concentration (MIC) values for ampicillin (>256 mg/L), meropenem (4–16 mg/L), ceftriaxone (>256 mg/L), and gentamicin (>256 mg/L) (Table 1). Growth at a concentration of ≥4 mg/L for meropenem is considered resistant. All the six CRVCs were found to harbor *bla*_NDM-1_ in the PCR assay.

**TABLE 1:** Antibiotic resistance profiles of donors, recipients, and their respective transconjugants^*b*^

Strain ID	Organism	Disc diffusion results														MIC (mg/L)				^*a*^Frequency of transfer
		AMP	CTX	CRO	CAZ	MEM	CHL	SXT	NA	NOR	OFX	STR	AZM	GEM	PB	MEM	AMP	CRO	GM	
AKM-1	*V. cholerae* O1 (donor)	R	R	R	R	R	I	R	R	S	S	R	S	R	S	16	>256	>256	>256	NA
AKM-2	*V. cholerae* O1 (donor)	R	R	R	R	R	S	R	R	S	S	R	S	R	S	8	>256	>256	>256	NA
AKM-3	*V. cholerae* O1 (donor)	R	R	R	R	R	I	R	R	S	S	R	S	R	S	16	>256	>256	>256	NA
AKM-4	*V. cholerae* O1 (donor)	R	R	R	R	R	S	R	R	S	S	R	S	R	S	16	>256	>256	>256	NA
AKM-5	*V. cholerae* O1 (donor)	R	R	R	R	R	S	R	R	S	S	R	S	R	S	16	>256	>256	>256	NA
AKM-6	*V. cholerae* O1 (donor)	R	R	R	R	R	S	R	R	S	S	R	S	R	S	4	>256	>256	>256	NA
J53	*E. coli* (recipient)	S	S	S	S	S	S	S	S	S	S	S	S	S	ND	0.032	0.125	0.075	0.19	NA
TC-J53	*E. coli* (transconjugant)	R	R	R	R	R	S	S	S	S	S	S	S	R	ND	8	>256	>256	>256	2.9 × 10^−2^
K12929	*V. parahaemolyticus* (recipient)	R	S	S	S	S	S	ND	S	S	S	I	S	S	ND	0.047	24	0.50	0.125	NA
TC-K12929	*V. parahaemolyticus* (transconjugant)	R	R	R	R	R	S	ND	S	S	S	I	S	R	ND	12	>256	>256	>256	1.22 × 10^−3^
IDH 6498	*Shigella flexneri* 4 (recipient)	R	S	S	S	S	ND	R	S	ND	ND	R	ND	S	ND	0.064	>32	0.38	0.125	NA
TC-6498	*Shigella flexneri* 4 (transconjugant)	R	R	R	R	R	ND	R	S	ND	ND	R	ND	R	ND	3	>256	>256	>256	2.35 × 10^−4^
IDH 3291/AKM-7	*V. cholerae* O1 (recipient)	R	S	S	S	S	S	R	R	S	S	R	S	S	R	0.25	4	0.47	0.38	NA
TC- 3291/AKM-8	*V. cholerae* O1 (transconjugant)	R	R	R	R	R	S	R	R	S	S	R	S	R	R	16	>256	>256	>256	1.4 × 10^−5^
BCH 0645	*Salmonella enterica* (recipient)	R	S	S	S	S	S	S	R	R	R	S	S	S	ND	0.064	>256	0.25	0.25	NA
TC-0645	*Salmonella enterica* (transconjugant)	R	R	R	R	R	S	S	R	R	R	S	S	R	ND	4	>256	>256	>256	1.42 × 10^−6^

^*a*^ Frequency of transfer was calculated as the number of transconjugants per donor cell.

^*b*^ AMP, ampicillin; CTX, cefotaxime; CRO, ceftriaxone, CAZ, ceftazidime; GM, gentamicin; MEM, meropenem; NA, nalidixic acid; SXT, sulfamethoxazole/trimethoprim; STR, streptomycin; C, chloramphenicol; NOR, norfloxacin; OFX, ofloxacin; AZM, azithromycin; PB, polymyxin B; S, sensitive; I, intermediate; R, resistant; ND, not done; NA, not applicable; MIC, minimal inhibitory concentration.

### WGS analysis of CRVC

WGS identified all CRVC belonging to sequence type ST-69 and revealed a ~4 Mb genome with a GC content of 47.6%. All the CRVC isolates revealed the presence of several ARGs, including *bla*_NDM-1_, *bla*_CMY-6_, and *bla*_DHA-7_, which confer resistance to β-lactams. Resistance to aminoglycosides was linked to the presence of genes, such as *strA*, *strB*, *rmtC*, and *aac(6')-Ib3*, while resistance to sulfonamides and trimethoprim was associated with *sul1*, *sul2*, and *dfrA1. floR* and *catB9* are responsible for chloramphenicol resistance. The WGS analysis also underscored a strong correlation between phenotypic and genotypic resistance. Most of these ARGs are associated with various MGEs, including plasmids, insertion sequences, and transposons.

### Phylogenetic clustering of the CRVC in the global context

**Fig 1:**
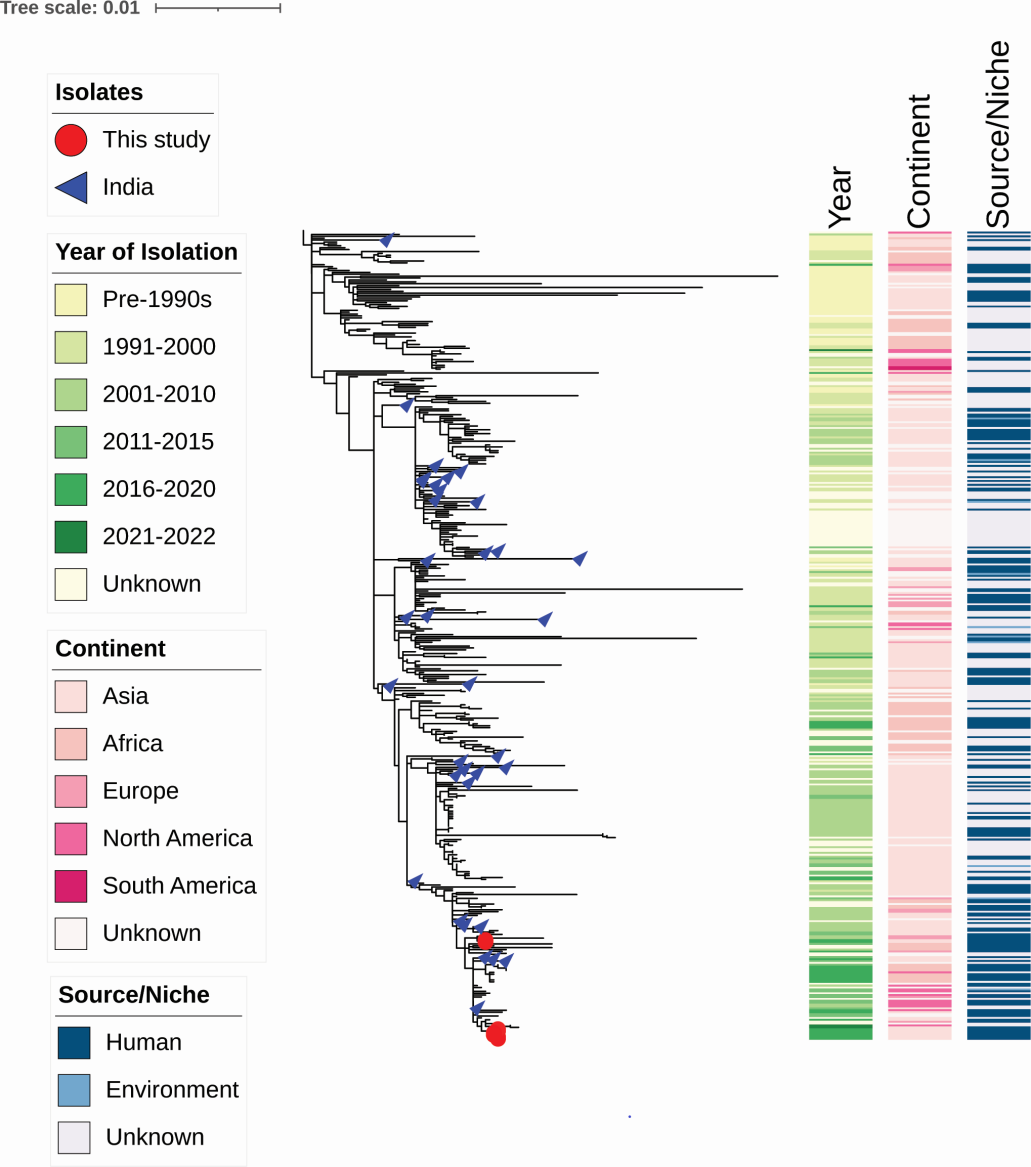
Maximum likelihood phylogenetic analysis was performed based on the core genome SNPs across 427 *V*. *cholerae* genomes, including the isolates from this study. The study isolates are marked in red, highlighting their position within the global collection. The accompanying color bars represent the year of isolation, continent, and source, with the codes explained in the figure inset. The tree scale denotes the number of substitutions per genome per site, indicating the evolutionary distance between the isolates analyzed.

Figure 1 presents the phylogenetic analysis of 426 VC genomes, including those from the current study. This collection encompasses a wide range of VC serogroups, with the tested isolates closely placed. Notably, the CRVC isolates (AKM-1 to AKM-6) formed a distinct cluster. Although AKM-7 (recipient VC) and AKM-8 (transconjugant VC) clustered together, they were slightly dissociated from the CRVC cluster, indicating their genetic difference. Moreover, a comparative genome analysis revealed no chromosomal SNPs between *V. cholerae* O1 transconjugant and its respective recipient. Additionally, the phylogenetic tree reveals that the clustering of VC isolates aligns with their year of isolation, geographic origin, and source. The test VC isolates clustered closely with genomes previously reported from India, reinforcing their regional origin, irrespective of their AMR profile. Within the CR isolates (AKM-1 to AKM-6), no appreciable genetic variation was observed, indicating they form a single clone with no detectable SNP differences.

### Characterization of the IncA/C plasmid carrying *bla*_NDM-1_

**Fig 2:**
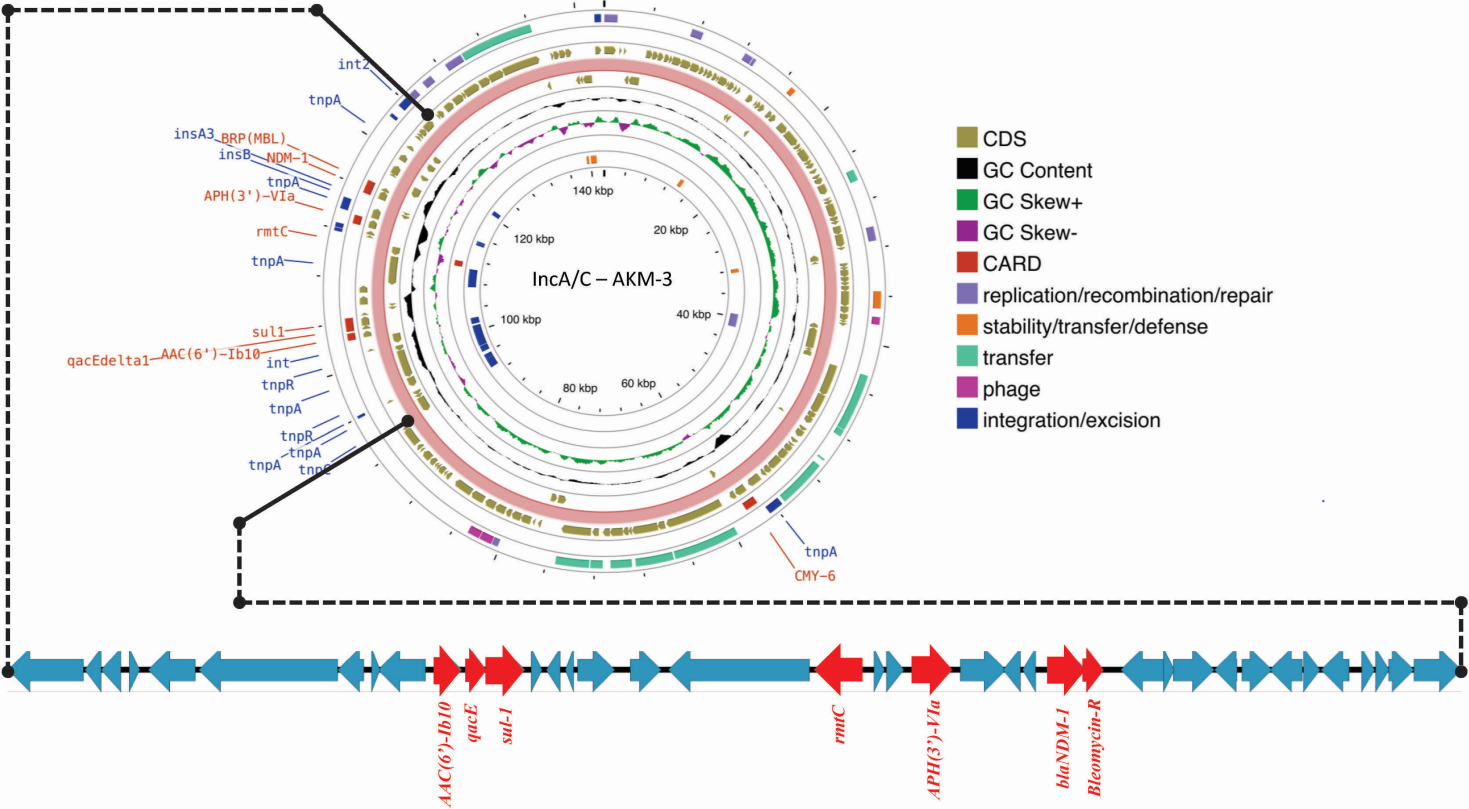
The circular representation of plasmid pNDM-VC identified in this study highlights key genomic features. The region encoding transposable elements, including the AMR gene cassette, is zoomed in to illustrate the genetic arrangement in detail. The GC content and the GC skew (+/−) are presented to emphasize hotspots, which show variation in the GC content due to the presence of mobile genetic elements carrying AMR genes. Additionally, genes associated with plasmid functions, such as replication, recombination/repair, stability, transfer, defense, phage-related elements, and integration/excision, are mapped across the plasmid sequence. This figure was generated using the Proksee server.

The assembled genome revealed a single plasmid, pNDM- *V. cholerae* O1, approximately 142 kb in size with an average GC content of 51.4%, encompassing 174 predicted coding sequences (CDSs). This plasmid belongs to sequence type 1 (ST-1) of the IncA/C plasmid group characterized by typical IncA/C replicons with key genes, such as *parA*, *parB*, *repA,* and *A053*. The IncA/C plasmid encodes multiple ARGs across several antimicrobial classes, including *bla*_NDM-1_, *bla*_CMY-6_, *bla*_DHA-7_, *aph(3')-VI*, *aph(3")-Ib*, *aph(6)-Id*, *aac(6')-Ib3*, *rmtC*, *sul1*, and *ble*_MBL_. The first region contains a cluster of ARGs, including *sul1*, *aac(6')-Ib3*, *aph(3')-VI*, *aph(3")-Ib*, *aph(6)-Id*, *rmtC*, *ble*_MBL_, *bla*_NDM-1_, and *qacE*, located between integrons (3′ conserved sequence of class I integron) and an IS26 sequence. The presence of the *traGH* conjugal transfer genes upstream in this region plays a crucial role in the horizontal transfer of the plasmid. The second region, which includes *bla*_CMY-6_, is flanked by a *tnpA* transposase and the type IV secretion system (T4SS) cluster, which contains additional conjugal transfer (*tra*) genes (Fig. 2).

### Comparative analysis of IncA/C plasmids revealed a diverse genetic background for *bla*_NDM-1_

**Fig 3:**
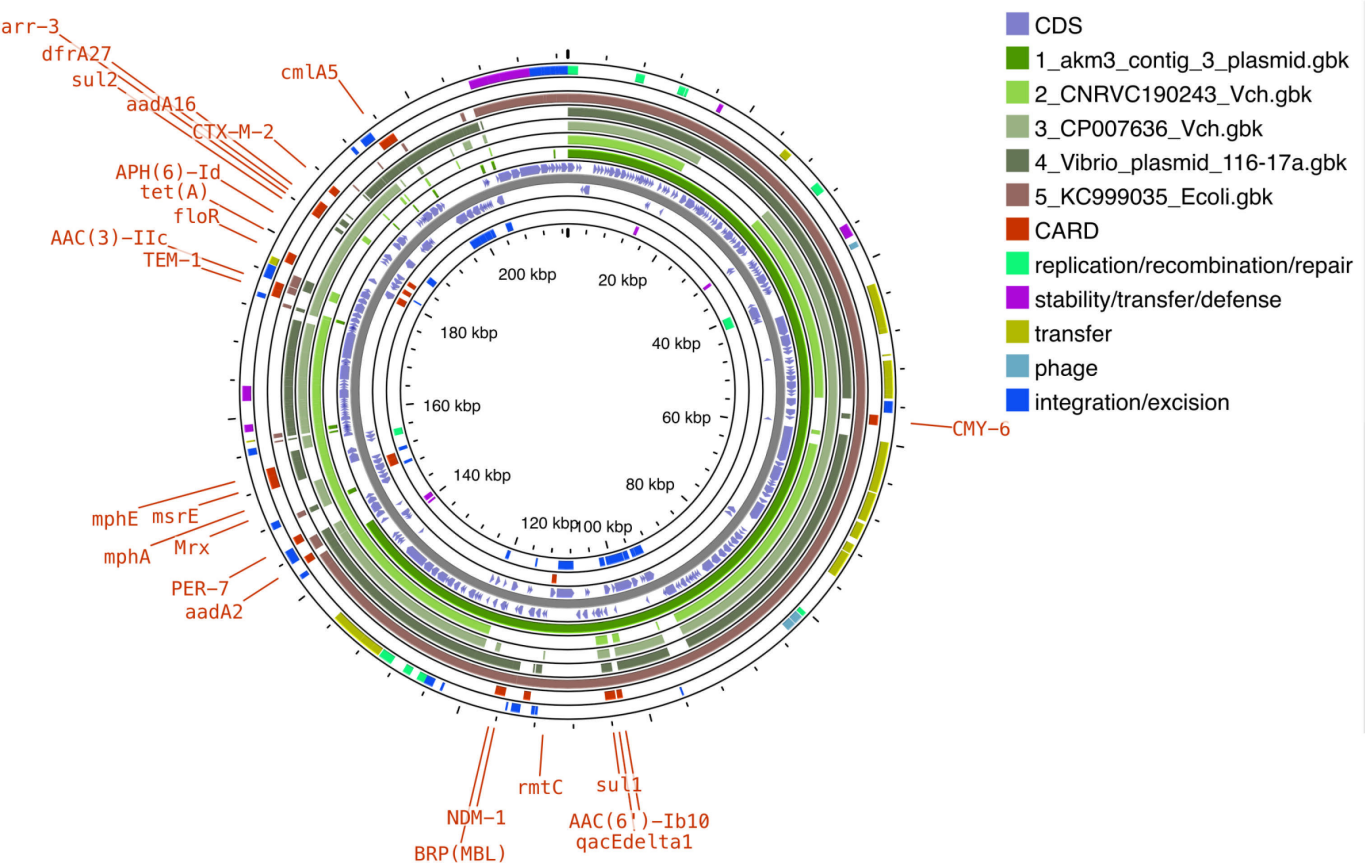
The circular representation of plasmid pNDM-VC was compared against closely related sequences obtained from BLAST analysis. A pan-genome backbone was constructed using all the plasmid sequences, which served as the reference for aligning and blasting individual plasmids. This comparison highlighted both similarities and differences, particularly in regions encoding MGEs and AMR genes, despite the plasmids sharing a similar backbone. The pan-genome alignment was generated using CGView-Pangenome, allowing for a detailed comparison of the genetic content across the plasmids.

**Fig 4:**
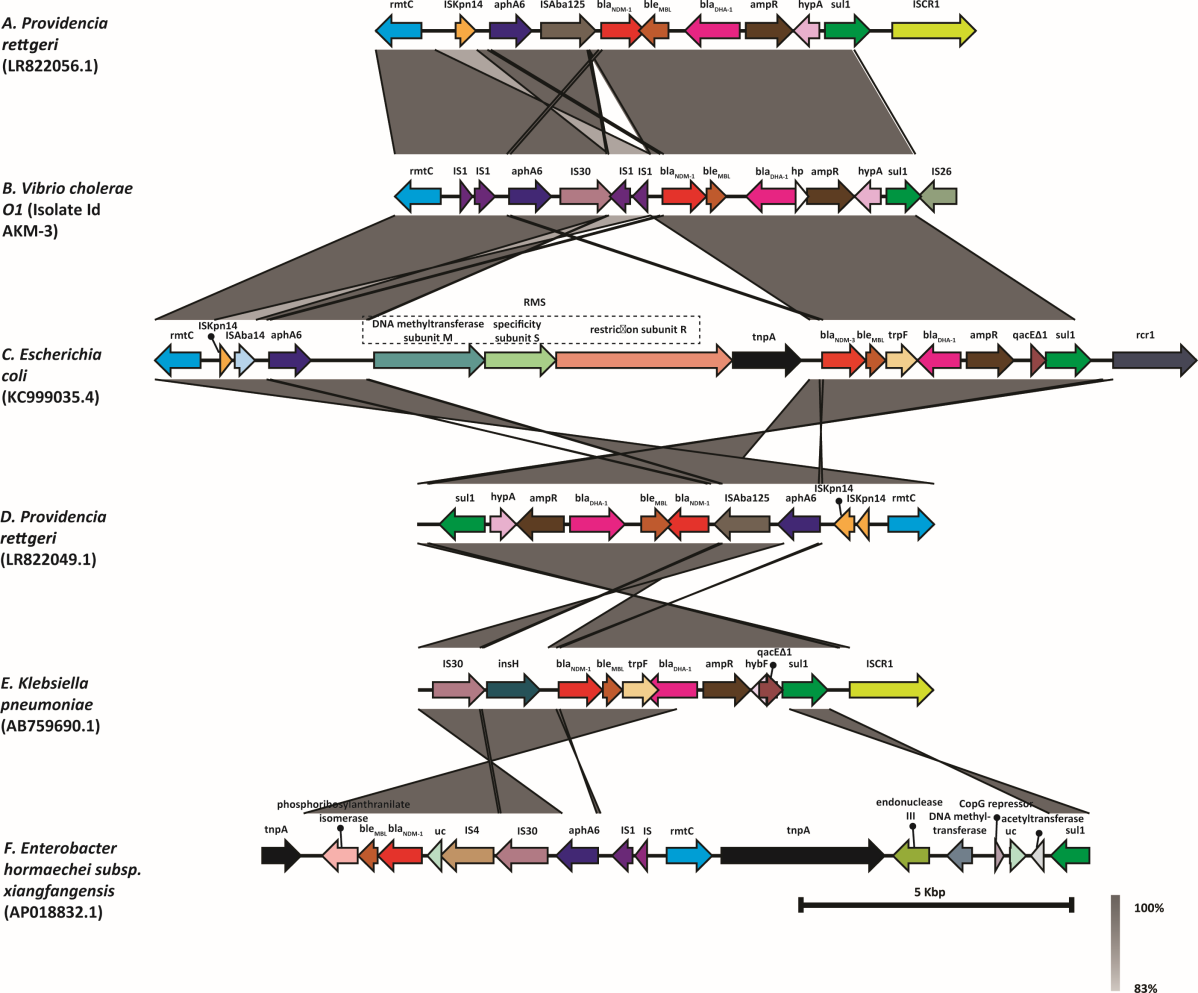
The linear representation illustrates the genetic arrangements of *bla*_NDM_-bearing mobile genetic elements across various bacterial species from different origins, as revealed through the BLAST analysis. The sequence of pNDM-VC shared a high degree of similarity with that of *Providencia rettgeri*, whereas *E. coli* harbored additional flanking genes surrounding *bla*_NDM_, showcasing the genetic diversity among species. This variation emphasizes the dynamic nature of *bla*_NDM_-carrying elements across different bacterial hosts. The figure was generated using EasyFig to visualize these comparative genomic arrangements. RMS, type 1 restriction-modification system; hp, hypothetical protein; uc, uncharacterized

Comparative BLAST analysis showed that the pNDM- *V. cholerae* O1 shares high similarity with the plasmids of *E. coli* (KC999035) and *V. cholerae* (CP007636), with 99% identity and 98% query coverage for *E. coli* and 99% identity with 78% query coverage for *V. cholerae*. The presence of conjugal transfer genes and integrons adjacent to the ARGs indicates that these genes were likely acquired from other members of Enterobacteriaceae. The ARGs on the plasmid are organized into two distinct regions. Initially, the plasmid sequences of three *V. cholerae* genomes harboring the *bla*_NDM_ gene and an *E. coli* genome (KC999035) displayed the highest similarity to the pNDM- *V. cholerae* O1. As depicted in Fig. 3, a pan-genome backbone was assembled using five plasmid sequences, followed by BLAST to identify regions of similarity. Interestingly, plasmids found in *V. cholerae* genomes (excluding the study isolates) harbored additional ARG clusters encoding extended-spectrum β-lactamases (ESBLs) and aminoglycoside-modifying enzymes (AMEs) but not the *bla*_NDM_ (except for *Vibrio*_plasmid_116-17a). This highlights the differences in the ARG content between plasmids of the similar IncA/C type. Additionally, the ARGs of VC were found to be highly dynamic, indicating frequent genetic rearrangements. As shown in Fig. 4, the genetic environment surrounding the *bla*_NDM_ has very high similarity despite being present in distinct bacterial species. This suggests that horizontal transfer and constant maintenance of the AMR mechanism are necessary across different bacterial species.

### Transmission ability of the *bla_NDM-1_*-carrying IncA/C plasmid through conjugation

Conjugation studies confirmed the effective transfer of the plasmid containing the *bla*_NDM-1_ to all the tested transconjugants. A single plasmid band identical in size to that of the donor CRVC was detected in all transconjugants. These transconjugants exhibited reduced MICs to meropenem, ceftriaxone, and gentamicin compared to the wild-type CRVC (Table 1). The AMR profiles of the transconjugants demonstrated resistance to ampicillin, ceftriaxone, cefotaxime, ceftazidime, meropenem, and gentamicin, suggesting that the *bla*_NDM-1_ plasmid carries ARGs to these antimicrobials. The transfer frequency revealed that the plasmid was transferred to *E. coli* J53 at a frequency of 2.9 × 10⁻², followed by *V. parahaemolyticus* (1.22 × 10⁻³), *S. flexneri* (2.35 × 10⁻⁴), *V. cholerae* O1 (1.4 × 10⁻⁵), and *S. enterica* (1.42 × 10⁻⁶) (Table 1). Notably, the transfer of the NDM plasmid to *S. enterica* was less competent compared to the other organisms studied.

## DISCUSSION

The increase of AMR plays a crucial role in shaping the evolutionary path of *V. cholerae*, especially in the epidemiology of cholera. The El Tor biotype of *V. cholerae* O1 has evolved over time, emerging as an MDR pathogen capable of causing devastating outbreaks. A key historical example is the cholera outbreak in Tanzania (1977–1978) driven by tetracycline-resistant *V. cholerae* O1 carrying IncC plasmids (15). More recently, studies have shown that *V. cholerae* clinical isolates have developed resistance to nearly all commonly used antimicrobials, highlighting the pathogen’s genomic plasticity (12, 16). This genomic adaptation allows *V. cholerae* to survive in hostile environments by acquiring MGEs, which further contribute to the emergence of MDR and even extensively drug-resistant (XDR) bacteria (12).

The first documented CRVC carrying the *bla*_NDM-1_ gene was identified in 2012 in southern India, although WGS was not performed at the time (6). Since then, carbapenem resistance has become a global concern, with cases of AMR *Vibrio* species being reported across almost all continents. While carbapenem resistance in *Vibrio* spp. is generally below 10%, countries, such as Bangladesh, Nigeria, Uganda, and South Africa, have reported higher resistance levels (8). The escalation of intermediate/reduced susceptibility trend indicates that these bacteria are evolving toward more resistant phenotypes, posing a serious public health threat. For example, in South Africa’s Eastern Cape, susceptibility to carbapenems has steadily declined over the past decade, underscoring the growing global issue of carbapenem-resistant *Vibrio* spp. (17). This trend elicits a major concern, as carbapenems have been reserved as last-line antimicrobials.

In our study, 50 clinical *V. cholerae* O1 isolates were phenotypically classified as an El Tor biotype with a PB-sensitive phenotype consistent with our previous findings (18, 19). These isolates carried the hypervirulent Haitian virulence gene alleles *ctxB7* and *tcpA^CIRS^*, along with a non-functional *rtxA* gene, similar to the Gujarat VC O1 serogroup identified during 2018 (18). Although tetracycline resistance is commonly reported in *V. cholerae* (20), none of the isolates in this study exhibited resistance to tetracycline. However, 12% (6/50) of the isolates displayed resistance to meropenem due to the presence of the *bla*_NDM-1_. These CRVC also demonstrated resistance to other antimicrobials, including nalidixic acid, streptomycin, trimethoprim/sulfamethoxazole, third-generation cephalosporins, and aminoglycosides. In the cgMLST, these strains were characterized as ST69, which is a common sequence type identified in all the 7P *V*. *cholerae* strains (21, 22). All the CRVC isolates are clustered together in the phylogenetic tree, indicating high genetic similarity with no detectable SNP differences among them. In addition, the inclusion of the recipient (AKM-7) and transconjugant (AKM-8) El Tor *V. cholerae* clinical isolates in the phylogeny further assesses whether the acquisition of the large-sized plasmid (142kbp pNDM-VC) led to any SNP-level changes in the host genome (i.e., any potential mutations following plasmid transfer) and verify that the plasmid was fully and correctly transferred. The distinct positioning of AKM-7 and AKM-8 from the CRVC isolates (AKM-1 to AKM-6) highlights the genetic diversity between carbapenem-susceptible and -resistant isolates. The close phylogenetic placement of AKM-7 and AKM-8 further suggests that the plasmid acquisition by AKM-8 (a recipient of AKM-7) did not result in any detectable chromosomal mutations. This likely reflects the experimental structure, where AKM-8 was derived from an overnight conjugation event, followed by a single sub-culture, limiting the opportunity for compensatory mutations to arise. It is possible that extended sub-culturing over multiple generations could have led to genomic adaptations to maintain the plasmid, but exploring such long-term evolutionary consequences was beyond the scope of the current study.

The *bla*_NDM-1_-carrying *V. cholerae* O1 isolates also carried *bla*_DHA-7_*, bla*_OXA-30_, and *bla*_CMY-6_. All the CRVCs harbored an IncA/C plasmid known for conferring resistance to multiple drug classes and its ability to transfer between bacterial species. This IncA/C plasmid has been transferred to many enteric bacteria, which express a different combination of MDR profiles. This observation suggests the important role of conjugative plasmids in the spread of AMR. Plasmids carrying carbapenemase encoding genes, such as *bla*_NDM-1_, are often associated with incompatibility plasmid groups like IncA/C, IncF, IncR, IncX, and others, which facilitate the spread of MDR within different bacterial populations (23, 24). The ability of IncC plasmids to evade CRISPR-Cas systems in *V. cholerae* further contributes to their persistence and adaptation.

### Conclusion

The emergence of CRVC carrying the *bla*_NDM-1_ marks an alarming shift in the epidemiology of cholera. Our study reveals that these VC not only possess resistance to carbapenems but also express the MDR profile. The IncA/C plasmids that carry the *bla*_NDM-1_ are highly competent in spreading AMR across several enteric bacterial species, underscoring the importance of enhanced genomic surveillance. The prevalence of CRVC is a growing global concern, particularly in regions where the use of antimicrobials is unrestricted. To mitigate the spread of such trends, there is an urgent need for stringent AMR surveillance, effective antimicrobial stewardship, and robust infection control measures. Without prompt action, the rise of CRVC and other enteric pathogens could significantly compromise current treatment strategies and pose a severe public health threat.

## MATERIALS AND METHODS

### Collection and analysis of clinical *V. cholerae* isolates

A total of 50 clinical *V. cholerae* isolates were obtained from cholera patients admitted to the Sir Sayajirao General Hospital in Baroda, Gujarat, India between August and November 2019. These isolates were initially cultured in a thiosulfate-citrate-bile salts-sucrose (TCBS) medium (Eiken, Japan) and further sub-cultured in Luria-Bertani (LB) broth (BD BBL, USA) or on LB agar plates for the subsequent biochemical and serological analyses. To confirm the serogroup, a slide agglutination test was performed using polyvalent O1-specific antiserum (Denka Seiken, Japan), followed by tests with monovalent antisera specific to Ogawa/Inaba serotype. The classical and El Tor biotypes of strains were differentiated by hemolysis on sheep blood agar, the Voges-Proskauer (VP) test, and polymyxin B (PB) susceptibility assay as per the established protocol (18).

### Antimicrobial susceptibility testing

The antimicrobial susceptibility testing of the *V. cholerae* isolates was made using the Kirby-Bauer disc diffusion method on Mueller-Hinton agar. The antimicrobials tested include ampicillin (10 µg), tetracycline (30 µg), trimethoprim/sulfamethoxazole (1.25/23.75 µg), chloramphenicol (30 µg), nalidixic acid (30 µg), ciprofloxacin (5 µg), norfloxacin (10 µg), ofloxacin (5 µg), gentamicin (10 µg), streptomycin (10 µg), cefotaxime (30 µg), ceftriaxone (30 µg), ceftazidime (30 µg), meropenem (10 µg), azithromycin (10 µg), and doxycycline (30 µg) (Difco, B.D., USA). Minimal inhibitory concentrations (MICs) were determined using E-test gradient strips (bioMérieux, Marcy l’Étoile, France) for meropenem, ampicillin, gentamicin, and ceftriaxone. Results were interpreted according to the Clinical and Laboratory Standards Institute (CLSI) guidelines, with *Escherichia coli* strain ATCC 25922 serving as a control (25, 26).

### DNA extraction and detection of virulence genes

Genomic DNA was extracted from VC isolates using the phenol-chloroform method and diluted to the required concentration for further analysis. The DNA served as a template for the mismatch amplification mutation assay (MAMA)-PCR to screen the genetic background of three key virulence genes: *ctxB*, *tcpA*, and *rtxA* by using three primer sets (27–29). Control strains, including *V. cholerae* O1 O395 (Classical), N16961 (El Tor), and EL-1786 (Haitian), were used to confirm the results. For plasmid DNA extraction, overnight cultures were grown on selective medium, and plasmid DNA was isolated from donors, recipients, and transconjugants using the Kado and Liu method (30), followed by analysis through gel electrophoresis using 0.8% agarose. Transconjugants were grown on selective agar plates supplemented with ceftriaxone (5 µg/mL).

### Plasmid transfer via conjugation assay

To assess plasmid transfer, conjugation assays were performed with *V. cholerae* O1 wild-type isolates (AKM-1 to AKM-6) harboring the *bla*_NDM-1_ plasmid as donors. Sodium azide-resistant *E. coli* J53, as well as other enteric pathogens like *Shigella flexneri* 4a (IDH 6498), *Salmonella enterica* serovar Enteritidis (BCH 0645), *Vibrio parahaemolyticus* (K12929), and an NDM-1-negative PB-resistant *V. cholerae* O1 (AKM-7/IDH 3291), served as recipients. Overnight-grown cultures of donors and recipients' isolates were inoculated in LB broth with selective antimicrobials for the mid-log phase culture. Both cultures were then mixed in a 1:1 ratio in tubes containing LB broth with no antimicrobials and incubated overnight at 37°C in a static condition. After incubation, serial dilutions were prepared, and 100 µL of the diluted culture was spread on the plates containing ceftriaxone (5 µg/mL) plus 100 µg/mL of sodium azide supplemented with MacConkey agar for *E. coli* J53, while XLD and TCBS agar were used for the screening of *S. flexneri/S. enterica* and *V. cholerae/V. parahaemolyticus,* respectively, to obtain transconjugants. Subsequently, 50 μg/mL PB plus ceftriaxone (5 µg/mL)-supplemented TCBS plates were used for the screening of *bla*_NDM-1_ transconjugants of PB-resistant *V. cholerae* O1. In all cases, the donor and recipient suspensions were also diluted in phosphate buffer saline (PBS) and plated on selective plates for each donor and recipient to confirm the purity and colony counts. The presence of *bla*_NDM-1_ in the transconjugants was determined by conventional PCR assay, and the result was checked by using 2% agarose gel stained with ethidium bromide (31). The frequency of transfer was calculated for each recipient by the formula, number of transconjugants that appeared on selective medium/no. of donor bacteria present when conjugation was initiated (32).

### Whole-genome sequencing

Total genomic DNA from eight VC isolates (AKM-1 to AKM-8) was extracted using the QIAamp DNA Mini Kit (Qiagen, Hilden, Germany). Quality and quantity were estimated using a NanoDrop Lite spectrophotometer (Thermo Fisher Scientific, Delaware, United States) and visualized by electrophoresis on a 0.8% agarose gel. WGS was performed on the Illumina NovaSeq 6000 platform generating paired-end reads. For two isolates, AKM-3 and AKM-4, hybrid assemblies were performed using both Illumina short and long reads from Oxford Nanopore Technologies' MinION sequencer. The assemblies were validated using CheckM software (v1.1.3).

### Genome analysis and ARG identification

For the analytical parameters and flags, default settings were used for all the used tools, unless otherwise stated. For selection, the *Vibrio cholerae* genomes from the global collection were considered using the Dereplicator tool to identify a representative subset of genomes that maintains the overall phylogenetic topology of the full data set (~8,000 genomes). From this collection, approximately 6,000 genomes were identified as *V. cholerae* O1 serogroup by screening for the presence of the *rfbV* gene. These 6,000 genomes were then further dereplicated using the same tool to yield a final representative set of assemblies for downstream analysis (*n* = 427: 418 global genomes; 1 reference genome N16961, and eight study isolates).

Raw sequencing reads were *de novo* assembled using SPAdes (33), and genome annotation was carried out using the RAST server (https://rast.nmpdr.org/). Core-genome multilocus sequence typing (cgMLST) was performed using the Center for Genomic Research’s database, focusing on seven housekeeping genes to determine the sequence type of carbapenem-resistant *V. cholerae* isolates. ARGs were identified using the ResFinder web server (http://genepi.food.dtu.dk/resfinder).

### Plasmid analysis

Plasmid analysis was conducted using PlasmidFinder 1.3 available on the CGE server (https://cge.food.dtu.dk/services/PlasmidFinder/), with whole-genome sequencing data from the study isolates. Plasmid incompatibility types were determined using pMLST 2.0 (https://cge.food.dtu.dk/services/pMLST/). The MGEs were identified using the MGE Finder tool on the same platform (https://cge.food.dtu.dk/services/MobileElementFinder/). For the assembly of carbapenemase-harboring plasmids, plasmidSPAdes (34) was utilized. Comparative plasmid analysis was conducted against two reference plasmids using Bowtie2, and the results were visualized with CGView (https://proksee.ca/).

### Phylogenetic analysis

From the 8,000 assembled genomes downloaded from EnteroBase, 426 *V*. *cholerae* O1 genomes, including the recipient (AKM-7) and transconjugant (AKM-8) El Tor *V. cholerae* isolates, were selected for analysis using the dereplicator tool (https://github.com/rrwick/Assembly-Dereplicator). Genomic alignment was performed, mapping the genomes against the reference strain *V. cholerae* O1 El Tor N16961 using snippy (https://github.com/tseemann/snippy). SNPs were extracted and used to construct a phylogenetic tree with the GTRGAMMA model in the RAxML software (https://github.com/amkozlov/raxml-ng).

## Data Availability

The raw sequencing reads from this study have been deposited in the NCBI database under accession number PRJNA1225860.

## SUPPLEMENTAL MATERIAL

Fig. S1 aac.00740-25-s0001.pdf Antibiotic susceptibility profile of *V. cholerae* O1 isolates.

Supplemental material aac.00740-25-s0002.docx Supplemental figure legend.

ASM does not own the copyrights to Supplemental Material that may be linked to, or accessed through, an article. The authors have granted ASM a non-exclusive, world-wide license to publish the Supplemental Material files. Please contact the corresponding author directly for reuse.

## References

[1] Sack DA, Sack RB, Nair GB, Siddique AK. 2004. Cholera. Lancet 363:223–233. doi:10.1016/s0140-6736(03)15328-7 14738797

[2] World Health Organization (WHO). 2024. Weekly epidemiological record No 36 99:479–496. https://iris.who.int/handle/10665/378714.

[3] Ceccarelli D, Alam M, Huq A, Colwell RR. 2016. Reduced susceptibility to extended-spectrum β-lactams in Vibrio cholerae isolated in Bangladesh. Front Public Health 4:231. doi:10.3389/fpubh.2016.00231 27803895 PMC5067765

[4] Dengo-Baloi LC, Semá-Baltazar CA, Manhique LV, Chitio JE, Inguane DL, Langa JP. 2017. Antibiotics resistance in El Tor Vibrio cholerae 01 isolated during cholera outbreaks in mozambique from 2012 to 2015. PLoS One 12:e0181496. doi:10.1371/journal.pone.0181496 28792540 PMC5549693

[5] Taneja N, Samanta P, Mishra A, Sharma M. 2010. Emergence of tetracycline resistance in Vibrio cholerae O1 biotype El Tor serotype Ogawa from north India. Indian J Pathol Microbiol 53:865–866. doi:10.4103/0377-4929.72014 21045454

[6] Mandal J, Sangeetha V, Ganesan V, Parveen M, Preethi V, Harish BN, Srinivasan S, Parija SC. 2012. Third-generation cephalosporin-resistant Vibrio cholerae, India. Emerg Infect Dis 18:1326–1328. doi:10.3201/eid1808.111686 22840562 PMC3414027

[7] Lassalle F, Al-Shalali S, Al-Hakimi M, Njamkepo E, Bashir IM, Dorman MJ, Rauzier J, Blackwell GA, Taylor-Brown A, Beale MA, Cazares A, Al-Somainy AA, Al-Mahbashi A, Almoayed K, Aldawla M, Al-Harazi A, Quilici ML, Weill FX, Dhabaan G, Thomson NR. 2023. Genomic epidemiology reveals multidrug resistant plasmid spread between Vibrio cholerae lineages in Yemen. Nat Microbiol 8:1787–1798. doi:10.1038/s41564-023-01472-1 37770747 PMC10539172

[8] Goh JXH, Tan LT-H, Law JW-F, Khaw K-Y, Ab Mutalib N-S, He Y-W, Goh B-H, Chan K-G, Lee L-H, Letchumanan V. 2022. Insights into carbapenem resistance in Vibrio species: current status and future perspectives. Int J Mol Sci 23:12486. doi:10.3390/ijms232012486 36293339 PMC9604146

[9] Ghosh A, Ramamurthy T. 2011. Antimicrobials & cholera: are we stranded? Indian J Med Res 133:225–231. 21415499 PMC3089056

[10] Spagnoletti M, Ceccarelli D, Rieux A, Fondi M, Taviani E, Fani R, Colombo MM, Colwell RR, Balloux F. 2014. Acquisition and evolution of SXT-R391 integrative conjugative elements in the seventh-pandemic Vibrio cholerae lineage. mBio 5:e01356-14. doi:10.1128/mBio.01356-14 25139901 PMC4147863

[11] Carraro N, Rivard N, Ceccarelli D, Colwell RR, Burrus V. 2016. IncA/C conjugative plasmids mobilize a new family of multidrug resistance islands in clinical Vibrio cholerae non-O1/non-O139 isolates from haiti. mBio 7:e00509-16. doi:10.1128/mBio.00509-16 27435459 PMC4958241

[12] Verma J, Bag S, Saha B, Kumar P, Ghosh TS, Dayal M, Senapati T, Mehra S, Dey P, Desigamani A, Kumar D, Rana P, Kumar B, Maiti TK, Sharma NC, Bhadra RK, Mutreja A, Nair GB, Ramamurthy T, Das B. 2019. Genomic plasticity associated with antimicrobial resistance in Vibrio cholerae. Proc Natl Acad Sci USA 116:6226–6231. doi:10.1073/pnas.1900141116 30867296 PMC6442563

[13] Shanta AS, Islam N, Al Asad M, Akter K, Habib MB, Hossain MJ, Nahar S, Godman B, Islam S. 2024. Resistance and co-resistance of metallo-beta-lactamase genes in diarrheal and urinary-tract pathogens in Bangladesh. Microorganisms 12:1589. doi:10.3390/microorganisms12081589 39203431 PMC11356267

[14] Briet A, Helsens N, Delannoy S, Debuiche S, Brisabois A, Midelet G, Granier SA. 2018. NDM-1-producing Vibrio parahaemolyticus isolated from imported seafood. J Antimicrob Chemother 73:2578–2579. doi:10.1093/jac/dky200 29796645

[15] Towner KJ, Pearson NJ, Mhalu FS, O’Grady F. 1980. Resistance to antimicrobial agents of Vibrio cholerae E1 Tor strains isolated during the fourth cholera epidemic in the United Republic of Tanzania. Bull World Health Organ 58:747–751. 6975183 PMC2395989

[16] Kuma GK, Opintan JA, Sackey S, Opare D, Dongdem AZ, Aryee E, Antwi L, Ofosu-Appiah LH, Owusu-Okyere G. 2014. Antibiotic resistant patterns amongst clinical Vibrio cholerae O1 isolates from the greater accra region, Ghana-2013. Int J Infect Dis 21:80. doi:10.1016/j.ijid.2014.03.594

[17] Gxalo O, Digban TO, Igere BE, Olapade OA, Okoh AI, Nwodo UU. 2021. Virulence and antibiotic resistance characteristics of Vibrio isolates from rustic environmental freshwaters. Front Cell Infect Microbiol 11:732001. doi:10.3389/fcimb.2021.732001 34490150 PMC8416912

[18] Shaw S, Samanta P, Chowdhury G, Ghosh D, Dey TK, Deb AK, Ramamurthy T, Miyoshi SI, Ghosh A, Dutta S, Mukhopadhyay AK. 2022. Altered molecular attributes and antimicrobial resistance patterns of Vibrio cholerae O1 El Tor strains isolated from the cholera endemic regions of India. J Appl Microbiol 133:3605–3616. doi:10.1111/jam.15794 36000378

[19] Samanta P, Saha RN, Chowdhury G, Naha A, Sarkar S, Dutta S, Nandy RK, Okamoto K, Mukhopadhyay AK. 2018. Dissemination of newly emerged polymyxin B sensitive Vibrio cholerae O1 containing Haitian-like genetic traits in different parts of India. J Med Microbiol 67:1326–1333. doi:10.1099/jmm.0.000783 29927375

[20] Ahmadi MH. 2021. Global status of tetracycline resistance among clinical isolates of Vibrio cholerae: a systematic review and meta-analysis. Antimicrob Resist Infect Control 10:115. doi:10.1186/s13756-021-00985-w 34362438 PMC8343947

[21] Ramamurthy T, Mutreja A, Weill FX, Das B, Ghosh A, Nair GB. 2019. Revisiting the Global Epidemiology of Cholera in conjuction with the genomics of Vibrio cholerae. Front Public Health 7:203. doi:10.3389/fpubh.2019.00203 31396501 PMC6664003

[22] Jubyda FT, Nahar KS, Barman I, Johura F-T, Islam MT, Sultana M, Ullah W, Tasnim J, Biswas SR, Monir MM, George CM, Camilli A, Ahmed N, Ross AG, Clemens JD, Alam M. 2023. Vibrio cholerae O1 associated with recent endemic cholera shows temporal changes in serotype, genotype, and drug-resistance patterns in Bangladesh. Gut Pathog 15:17. doi:10.1186/s13099-023-00537-0 37046358 PMC10090749

[23] Rozwandowicz M, Brouwer MSM, Fischer J, Wagenaar JA, Gonzalez-Zorn B, Guerra B, Mevius DJ, Hordijk J. 2018. Plasmids carrying antimicrobial resistance genes in Enterobacteriaceae. J Antimicrob Chemother 73:1121–1137. doi:10.1093/jac/dkx488 29370371

[24] Kocsis E, Gužvinec M, Butić I, Krešić S, Crnek SŠ, Tambić A, Cornaglia G, Mazzariol A. 2016. blaNDM-1 carriage on IncR plasmid in Enterobacteriaceae Strains. Microb Drug Resist 22:123–128. doi:10.1089/mdr.2015.0083 26484384

[25] Bauer AW, Kirby WM, Sherris JC, Turck M. 1966. Antibiotic susceptibility testing by a standardized single disk method. Am J Clin Pathol 45:493–496. 5325707

[26] CLSI. 2020. Performance standards for antimicrobial susceptibility testing. In M100 ED30. Clinical and Laboratory Standard Institute, PA.

[27] Naha A, Pazhani GP, Ganguly M, Ghosh S, Ramamurthy T, Nandy RK, Nair GB, Takeda Y, Mukhopadhyay AK. 2012. Development and evaluation of a PCR assay for tracking the emergence and dissemination of haitian variant ctxB in Vibrio cholerae O1 strains isolated from Kolkata, India. J Clin Microbiol 50:1733–1736. doi:10.1128/JCM.00387-12 22357499 PMC3347119

[28] Ghosh P, Naha A, Basak S, Ghosh S, Ramamurthy T, Koley H, Nandy RK, Shinoda S, Watanabe H, Mukhopadhyay AK. 2014. Haitian variant tcpA in Vibrio cholerae O1 El Tor strains in Kolkata, India. J Clin Microbiol 52:1020–1021. doi:10.1128/JCM.03042-13 24371245 PMC3957741

[29] Ghosh P, Naha A, Pazhani GP, Ramamurthy T, Mukhopadhyay AK. 2014. Genetic traits of Vibrio cholerae O1 haitian isolates that are absent in contemporary strains from Kolkata, India. PLoS One 9:e112973. doi:10.1371/journal.pone.0112973 25415339 PMC4240540

[30] Kado CI, Liu ST. 1981. Rapid procedure for detection and isolation of large and small plasmids. J Bacteriol 145:1365–1373. doi:10.1128/jb.145.3.1365-1373.1981 7009583 PMC217141

[31] Shanthi M, Sekar U, Kamalanathan A, Sekar B. 2014. Detection of New Delhi metallo beta lactamase-1 (NDM-1) carbapenemase in Pseudomonas aeruginosa in a single centre in southern India. Indian J Med Res 140:546–550. 25488450 PMC4277142

[32] Sarkar A, Pazhani GP, Chowdhury G, Ghosh A, Ramamurthy T. 2015. Attributes of carbapenemase encoding conjugative plasmid pNDM-SAL from an extensively drug-resistant Salmonella enterica serovar Senftenberg. Front Microbiol 6:969. doi:10.3389/fmicb.2015.00969 26441902 PMC4569734

[33] Prjibelski A, Antipov D, Meleshko D, Lapidus A, Korobeynikov A. 2020. Using SPAdes de novo assembler. Curr Protoc Bioinformatics 70:e102. doi:10.1002/cpbi.102 32559359

[34] Antipov D, Hartwick N, Shen M, Raiko M, Lapidus A, Pevzner PA. 2016. plasmidSPAdes: assembling plasmids from whole genome sequencing data. Bioinformatics 32:3380–3387. doi:10.1093/bioinformatics/btw493 27466620

