# Supplementary figures and images for: Genomic portrayal of emerging carbapenem-resistant El Tor variant Vibrio cholerae O1
Source: Antimicrob Agents Chemother. 2025 Oct 17;69(12):e00740-25. doi: 10.1128/aac.00740-25 (PMC12691646; doi:10.1128/aac.00740-25)

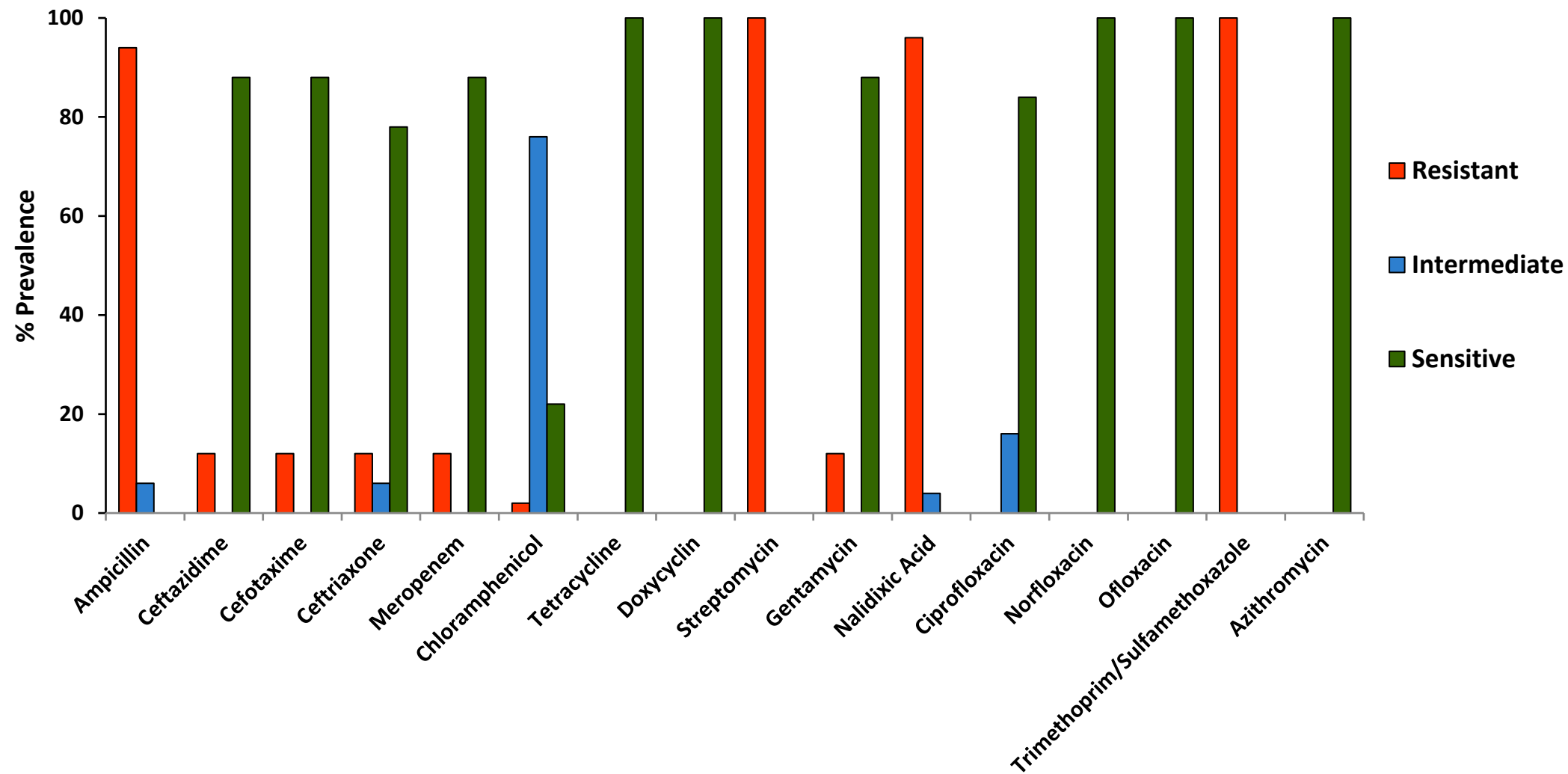

Supplement: Fig. S1 — Antibiotic susceptibility profile of V. cholerae O1 isolates. [file aac.00740-25-s0001.pdf]
