# Supplementary material for: Genomic portrayal of emerging carbapenem-resistant El Tor variant Vibrio cholerae O1
Source: Antimicrob Agents Chemother. 2025 Oct 17;69(12):e00740-25. doi: 10.1128/aac.00740-25 (PMC12691646; doi:10.1128/aac.00740-25)
Supplement: Supplemental material — Supplemental figure legend. [file aac.00740-25-s0002.docx]

Supplementary Figure 1

Antibiotic susceptibility profile of *V. cholerae* O1 isolates from Baroda, Gujarat determined by Kirby-Bauer disc diffusion method
